# Asymptotic stability of a modified Lotka-Volterra model with small immigrations

**DOI:** 10.1038/s41598-018-25436-2

**Published:** 2018-05-04

**Authors:** Takeru Tahara, Maica Krizna Areja Gavina, Takenori Kawano, Jerrold M. Tubay, Jomar F. Rabajante, Hiromu Ito, Satoru Morita, Genki Ichinose, Takuya Okabe, Tatsuya Togashi, Kei-ichi Tainaka, Akira Shimizu, Takashi Nagatani, Jin Yoshimura

**Affiliations:** 1 Department of Mathematical and Systems Engineering, Shizuoka University, 3-5-1 Johoku, Naka-ku, Hamamatsu, 432-8561 Japan; 2 Graduate School of Science and Technology, Shizuoka University, 3-5-1 Johoku, Naka-ku, Hamamatsu, 432-8561 Japan; 3 Mathematics Division, Institute of Mathematical Sciences and Physics, University of the Philippines Los Baños, College, Laguna, 4031 Philippines; 4 Graduate School of Integrated Science and Technology, Shizuoka University, 3-5-1 Johoku, Naka-ku, Hamamatsu, 432-8561 Japan; 5 Department of General Systems Studies, University of Tokyo, 3-8-1 Komaba, Meguro, Tokyo, 153-8902 Japan; 6 Marine Biosystems Research Center, Chiba University, Uchiura, Kamogawa, Chiba, 299-5502 Japan; 7 Department of Biological Sciences, Graduate School of Science and Engineering, Tokyo Metropolitan University, Hachioji, Tokyo, 192-0397 Japan; 8 Department of Mechanical Engineering, Shizuoka University, 3-5-1 Johoku, Naka-ku, Hamamatsu, 432-8561 Japan; 9 Department of Environmental and Forest Biology, State University of New York College of Environmental Science and Forestry, Syracuse, NY 13210 USA

## Abstract

Predator-prey systems have been studied intensively for over a hundred years. These studies have demonstrated that the dynamics of Lotka-Volterra (LV) systems are not stable, that is, exhibiting either cyclic oscillation or divergent extinction of one species. Stochastic versions of the deterministic cyclic oscillations also exhibit divergent extinction. Thus, we have no solution for asymptotic stability in predator-prey systems, unlike most natural predator-prey interactions that sometimes exhibit stable and persistent coexistence. Here, we demonstrate that adding a small immigration into the prey or predator population can stabilize the LV system. Although LV systems have been studied intensively, there is no study on the non-linear modifications that we have tested. We also checked the effect of the inclusion of non-linear interaction term to the stability of the LV system. Our results show that small immigrations invoke stable convergence in the LV system with three types of functional responses. This means that natural predator-prey populations can be stabilized by a small number of sporadic immigrants.

## Introduction

In the past, unexpected large quotas in animal and fish catches had been occasionally reported. The phenomenon was attributed to the predators in those communities^1–4^. This predator and prey relationship is probably the most studied ecological dynamics in recent history. Theoretical studies of this system began when Alfred Lotka and Vito Volterra independently developed the well-known predator-prey model in the 1920s. Lotka developed the model to study autocatalytic chemical reactions and Volterra extended it to explain the fish catches in the Adriatic Sea. Since the earliest developments of the basic Lotka-Volterra system (LV system)^5–10^, many mathematical variations of predator-prey systems have been developed to explain unexpected changes and temporal fluctuations in the dynamics of animal populations. This system is considered to explain the dynamics of natural populations of snowshoe hare (*Lepus americanus*, the prey) and Canadian lynx (*Lynx canadensis*, the predator) that were estimated from the yearly changes in the collected number of furs^11^. In addition, unlike in natural communities, it has been shown that long-term coexistence of predators and prey is possible in a laboratory using predatory and prey mites^12^. Thus, the LV system has become the classical mathematical model for explaining the predator-prey interactions in natural communities^5–11^.

Mathematical properties of the classical LV system show either cyclic oscillation or divergent extinction of one species^13^. In any closed LV system, it is also important to note that the predators will eventually die out with the extinction of the preys. This means that the persistent predator-prey systems, without additional stabilizing mechanisms, should exhibit cyclic oscillations. However, stable coexistence in wild predator-prey systems has been observed^10^. Nonetheless, the case of the lynx-hare interaction^8^ seems extremely unique natural predator-prey system. We often find stable coexistence of both prey and predator populations in the wild such as that of spider wasps (*Pompilidae* family, the predator) and spiders (the prey)^10^. These observations imply that there should be an additional stabilizing mechanism in natural predator-prey systems. For example, strong intraspecific competition in both predator and prey yields stable coexistence^14^. However, we have no evidence of such strong intraspecific competition in the wild. Thus, these observations of the most natural systems contradict with the original solution of the classical LV system.

In this paper, we explore the convergent solutions in predator-prey systems by modifying the classical LV system. Because most predator-prey systems in the wild are not isolated, we consider the effects of fixed (or random) number of immigrants at regular intervals on the predator and prey populations. By adding few immigrants, the LV systems with type I, II, and III functional responses exhibit asymptotic stability. Similarly, adding few immigrants to the predator population stabilizes the modified LV systems where both predator and prey coexist. In the latter case, the LV system may be interpreted as a host-parasite system, because the parasites are more likely to become immigrants. We then briefly discuss the implications of the modified LV system on the predator-prey systems found in the wild.

## Models and Results

Here, we consider the modified Lotka-Volterra systems with few predator and prey immigrants. Specifically, we analyze the asymptotic stability of the predator-prey systems by adding an immigration factor ***C(x)*** into the prey population or adding an immigration factor ***D(y)*** into the predator population in the classical LV system. The modified Lotka-Volterra systems with few immigrants is as follows:

1 $$\{\begin{array}{cc}\frac{dx}{dt}=rx-axy+C(x), & r,a\,{\rm{are}}\,{\rm{constant}}\\ \frac{dy}{dt}=bxy-my+D(y), & b,m\,{\rm{are}}\,{\rm{constant}}\end{array}$$

where ***x*** represents the prey population and *y* represents the predator population. The immigration function can be modeled into two ways:

2 $$C(x)=\{\begin{array}{cc}c, & c\ge 0\\ \frac{c}{x}, & c > 0\end{array}$$

where ***c*** represents the number of prey immigrants, ***c/x*** represents the proportion of prey immigrants. Similarly,

3 $$D(y)=\{\begin{array}{cc}d, & d\ge 0\\ \frac{d}{y}, & d > 0\end{array}$$

where ***d*** represents the number of predator immigrants, ***d/y*** represents the proportion of predator immigrants. We used the following assumptions for equations (2) and (3): *x* ≠ 0 (or *y* ≠ 0) when *C*(*x*) = *c*/*x* (or *D*(*y*) = d/*y*). In this model, the parameters ***r***, ***b*** represent the reproduction rate of prey and the birth rate of predator for each prey captured, respectively. Parameters ***a*** and ***m*** represent the rate at which predators consume the prey and the mortality rate of predators, respectively.

Here, we consider the following four cases of immigration factor to investigate its effect to the long-term population dynamics of the predator-prey system (1):

(Case A1) prey immigrants (i.e., *C(x)* = *c*, *D(y)* = 0)

(Case B1) predator immigrants (i.e., *C(x)* = 0, *D(y)* = *d*)

(Case C1) few prey immigrants (i.e., *C(x)* = *c*/*x*, *D(y)* = 0)

(Case D1) few predator immigrants (i.e., *C(x)* = 0, *D(y)* = *d*/*y*)

We also consider the following four cases of the migration factor:

(Case A2) prey migrants (i.e., *C(x)* = −*c*, *D(y)* = 0)

(Case B2) predator migrants (i.e., *C(x)* = 0, *D(y)* = −*d*)

(Case C2) few prey migrants (i.e., *C(x)* = −*c*/*x*, *D(y)* = 0)

(Case D2) few predator migrants (i.e., *C(x)* = 0, *D(y)* = −*d*/*y*).

Analytically, we investigate the asymptotic stability of a steady state solution for the eight cases of the modified Lotka-Volterra systems with few immigrants (or migrants) into prey or predator population. We found that the immigration into the predator population has the same stabilization effect as that of the prey (refer to the supporting text in the Supplementary Information for the analytical solution). Moreover, we found that the immigration of both prey and predator (i.e., *C(x)* = *c*, *D(y)* = *d*) also stabilizes LV system (1). However, migration destabilizes the system for both populations **(**refer to the supporting text in the Supplementary Information for the analytical solution). The stability analysis of the steady-state solution of the modified LV system with small immigration is shown below:

Case A1: Asymptotic stability of the LV system with few prey immigrants **(**refer to the supporting text in the Supplementary Information for the analytical solution). Here we investigate the stability of a steady state solution $({x}^{\ast },{y}^{\ast })=(\frac{m}{b},\frac{mr+bc}{am})$ of the modified LV system (1) with few prey immigrants (i.e., *C(x)* = *c*, *D(y)* = 0). We have shown that the coexistence equilibrium $({x}^{\ast },{y}^{\ast })=(\frac{m}{b}\,\frac{mr+bc}{am})$ is asymptotically stable since the rate of amplitude decay $\gamma =\frac{bc}{2m} > 0$, for all *b*, *m*>0.

Case B1: Asymptotic stability of the LV system with few predator immigrants **(**refer to the supporting text in the Supplementary Information for the analytical solution**)**. Here we investigate the stability of a steady state solution $({x}^{\ast },{y}^{\ast })=\,(\frac{mr-ad}{br},\frac{r}{a})$ of the modified LV (1) system with few predator immigrants (i.e., *C(x)* = 0, *D(y)* = *d*). We have shown that the coexistence equilibrium $\,({x}^{\ast },{y}^{\ast })=(\frac{mr-ad}{br},\frac{r}{a})$ where *mr* > *ad* is asymptotically stable since the rate of amplitude decay $\gamma =\frac{ad}{2r} > 0$, for all *a*, *r* > 0.

Case C1: Asymptotic stability of the LV system with few constant prey immigrants (refer to the supporting text in the Supplementary Information for the analytical solution**)**. Here we investigate the stability of a steady state solution $\,({x}^{\ast },{y}^{\ast })=(\frac{m}{b},\frac{{m}^{2}r+{b}^{2}c}{a{m}^{2}})$ of the modified LV system (1) with few prey constant immigrants (i.e., *C(x)* = *c/x*, *D(y)* = 0). We have shown that the coexistence equilibrium $\,({x}^{\ast },{y}^{\ast })=(\frac{m}{b},\frac{{m}^{2}r+{b}^{2}c}{a{m}^{2}})$ is asymptotically stable since the rate of amplitude decay $\gamma =\frac{{b}^{2}c}{{m}^{2}} > 0$, for all *b*, *m* > 0.

Case D1: Asymptotic stability of the LV system (1) with few constant predator immigrants **(**refer to the supporting text in the Supplementary Information for the analytical solution**)**. Here we investigate the stability of a steady state solution $\,({x}^{\ast },{y}^{\ast })=(\frac{m{r}^{2}-{a}^{2}d}{b{r}^{2}},\frac{r}{a})$ where *mr*^2^ > *a*^2^*d* of the modified LV system with few constant predator immigrants (i.e., *C(x)* = 0, *D(y)* = *d*/*y*). We have shown that the coe*x*istence equilibrium $\,({x}^{\ast },{y}^{\ast })=(\frac{m{r}^{2}-{a}^{2}d}{b{r}^{2}},\frac{r}{a})$ is asymptotically stable since the rate of amplitude decay $\gamma =\frac{{a}^{2}d}{{r}^{2}} > 0$, for all *a*, *r* > 0.

**Figure 1:**
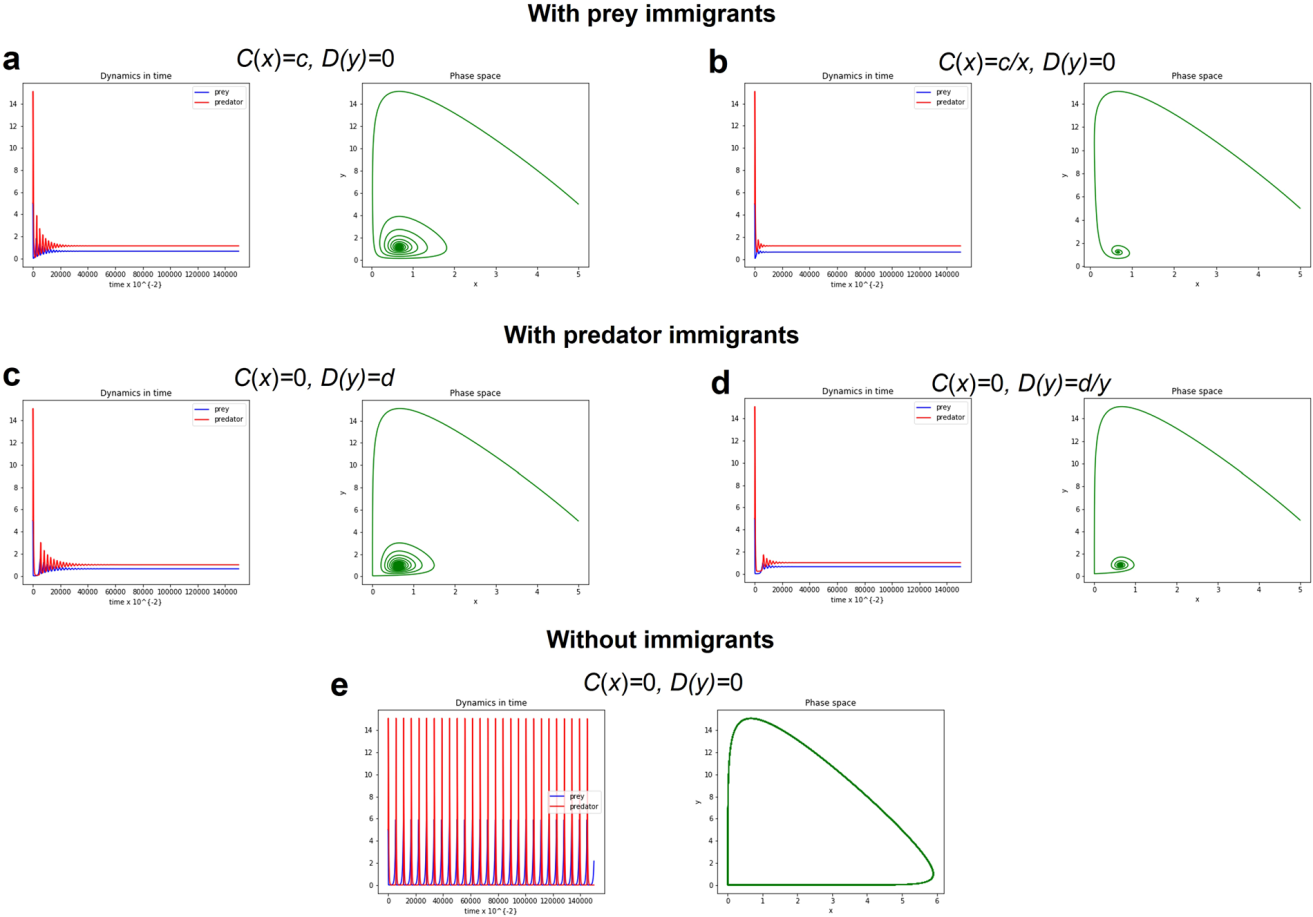
Phase space and long-term population dynamics of the predator-prey population described by LV system (1) with type I functional response and with/without small immigrants. Four cases of the modified LV system (1) with small immigration is compared to the classical LV system. Parameter values: Initial *x* = 5, Initial *y* = 5, *r* = 0.1, *a* = 0.1, *b* = 0.3, *m* = 0.2, *c* = 0.01, *d* = 0.01.

**Figure 2:**
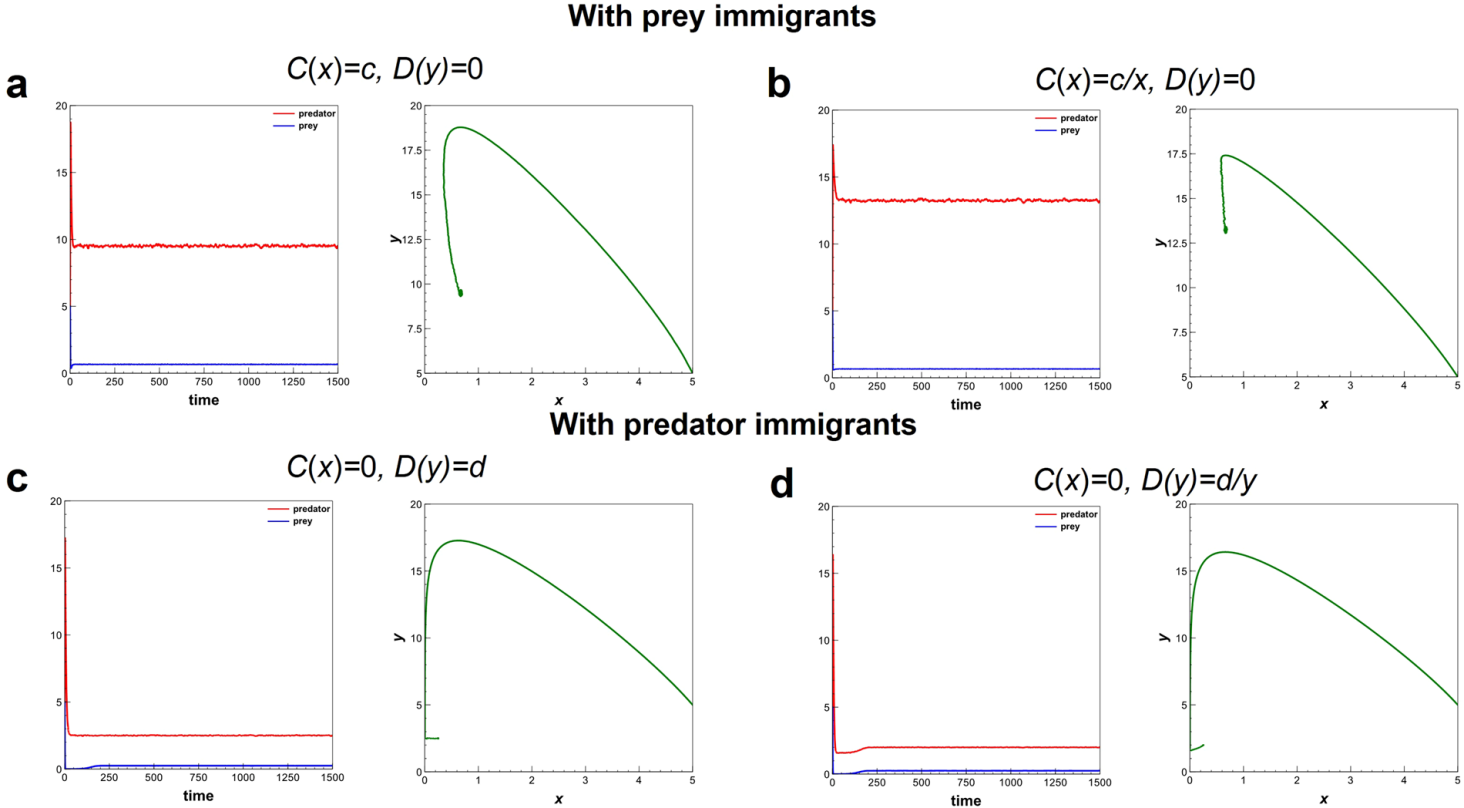
Modified LV model with random number of immigration at regular interval. Four cases of the modified LV system (1) with random number of immigration. Parameter values: Initial *x* = 5, Initial *y* = 5, *r* = 0.2, *a* = 0.1, *b* = 0.3, *m* = 0.2, *c* = random(0.001, 1), *d* = random(0.001, 1). Results are shown for 1,500 steps, with an average of 15 runs. Random (*i*, *j*) means uniform random number between *i* and *j*.

Using computer simulation, we examine the dynamics in the LV system (1) which can be seen in Figs 1–2. We illustrate sample trajectories of LV system (1) where *C*(*x*) = *c, D(y)* = 0 (Figs 1a and 2a), *C*(*x*) = *c/x, D(y)* = 0 (Figs 1b and 2b), *C*(*x*) = 0*, D(y)* = *d* (Figs 1c and 2c) and *C*(*x*) = 0*, D(y)* = *d/y* (Figs 1d and 2d). The resulting dynamics of the LV systems with the inclusion of a fixed number of immigrants on the predator or prey populations stabilizes the system (Fig. 1, *c*,*d* = 0.01), compared to the result of the classical LV systems which exhibits periodic oscillation (Fig. 1e, *c,d* = 0). However, the resulting dynamics with the inclusion of a positive migration factor destabilizes the system, i.e., no positive stable equilibria (see Supplementary Text for the stability analysis). In addition, LV system (1) with random number of immigration at regular interval will also exhibit convergence (Fig. 2, *c,d* = random(0.001, 1)) while few immigrants will lead to asymptotic stability of the system (Fig. 1). Introducing a random number of immigrants in the LV system will only reduce the magnitude of oscillation but will still stabilize the system (Fig. 2). Note that also random immigration has the weakest effect on the population dynamics of the predator-prey model (Fig. 2). Moreover, as long as *c* and *d* are positive, their magnitudes do not affect the outcome.

We also used the modified LV model with non-linear functional response and small immigrants. We change the predation term using a general (non-linear) functional response. The modified LV model is shown on the following equations^6,7,10^:

4 $$\{\begin{array}{cc}\frac{dx}{dt}=rx-\frac{a{x}^{1+\alpha }\,y}{1+h{x}^{1+\alpha }}+C(x), & h,r,a\,{\rm{are}}\,{\rm{constant}}\\ \frac{dy}{dt}=\frac{b{x}^{1+\alpha }\,y}{1+h{x}^{1+\alpha }}-my+D(y), & h,b,m\,{\rm{are}}\,{\rm{constant}}\end{array}$$

where *h, α* are the functional response coefficient which involve handling time etc. and Hill exponent, respectively. The values for *C*(*x*) and *D*(*y*) are similar to equations (2) and (3). Note that when *h* = 0 and α = 0 then the interaction term is referred to as type I functional response which is equivalent to LV system (1). When *h* ≠ 0 and α = 0 then the interaction term is referred to as type II functional response. Moreover, when *h* ≠ 0 and α > 0 then the interaction term is referred to as type III functional response. To be more specific we use α = 1 for the type III functional response.

**Figure 3:**
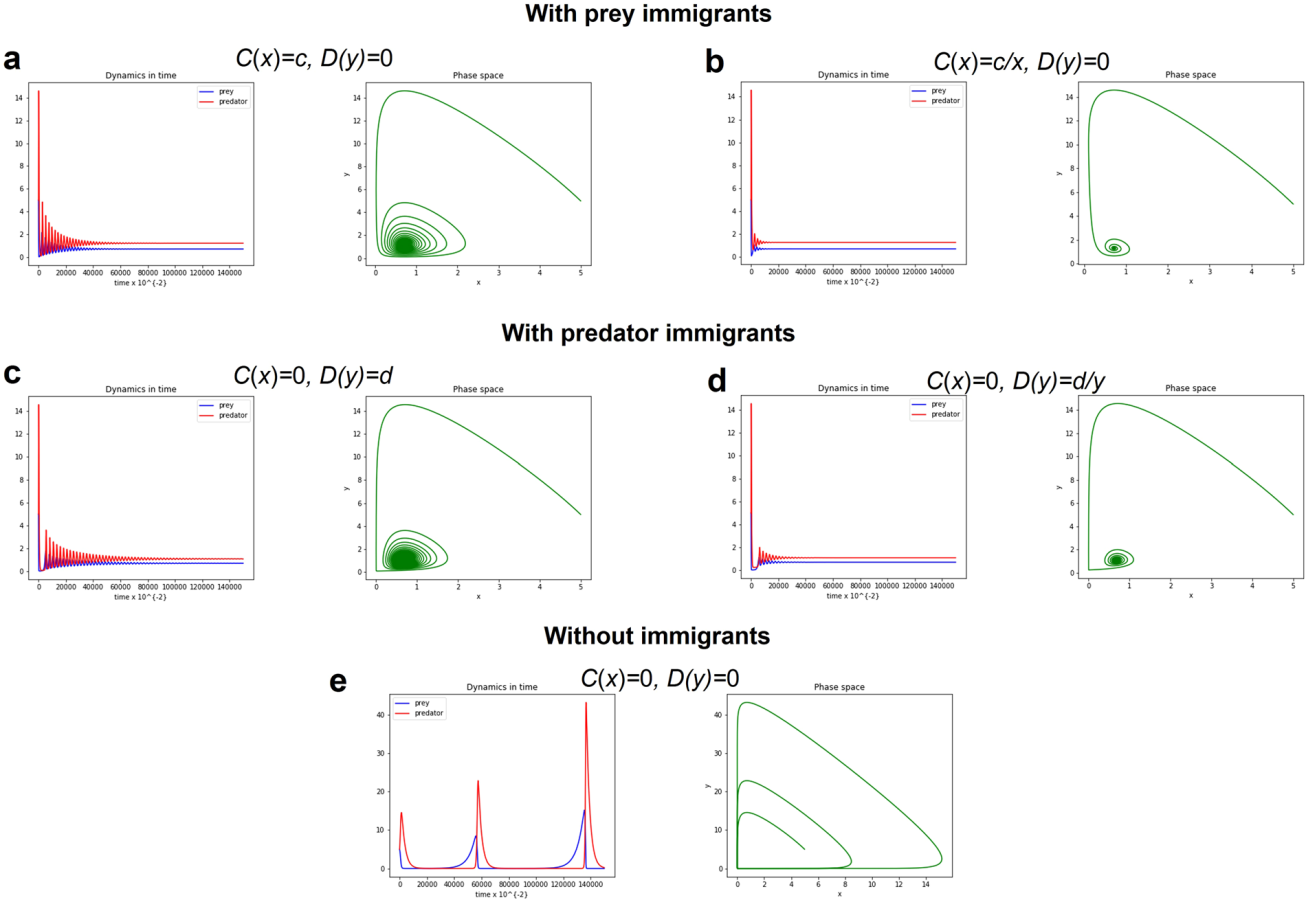
Phase space and long-term population dynamics of the predator-prey population described by LV system (4) with type II functional response and with/without small immigrants. Four cases of the modified LV system (4) with small immigration is compared to LV system (4) without small immigrants. Parameter values: Initial *x* = 5, Initial *y* = 5, *r* = 0.1, *a* = 0.1, *b* = 0.3, *m* = 0.2, *c* = 0.01, *d* = 0.01 and *h* = 0.1.

**Figure 4:**
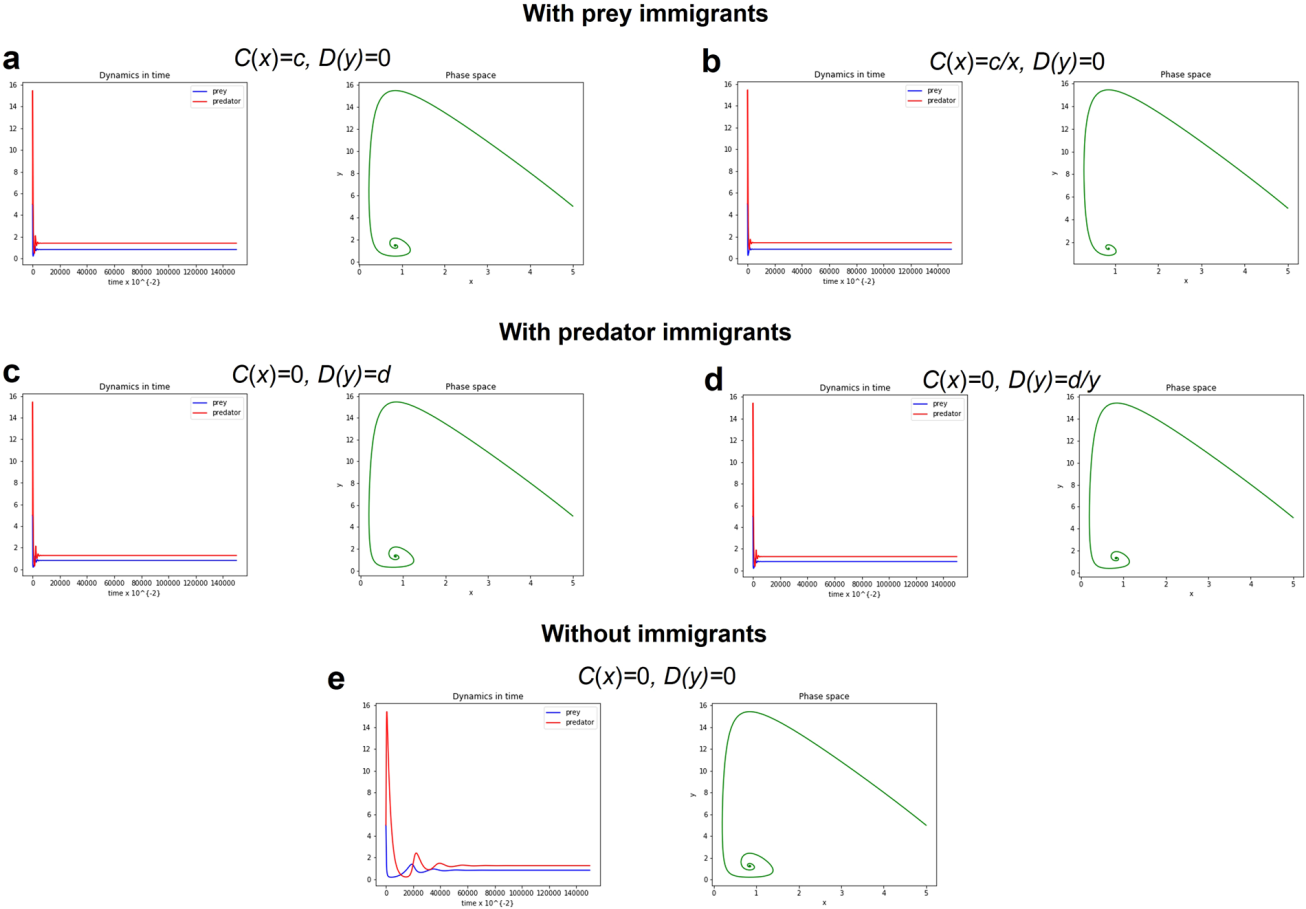
Phase space and long-term population dynamics of the predator-prey population described by LV system (4) with type III functional response and with/without small immigrants. Four cases of the modified LV system (4) with small immigration is compared to LV system (4) without small immigrants. Parameter values: Initial *x* = 5, Initial *y* = 5, *r* = 0.1, *a* = 0.1, *b* = 0.3, *m* = 0.2, *c* = 0.01, *d* = 0.01 and *h* = 0.1.

We summarize the nature of equilibria of the modified LV system (4) in Table 1 (refer to the Supplementary Information for the analytical solution). Satisfying the condition to become locally asymptotically stable (refer to Supplementary Tables S2–S5), we show illustrations for the LV system (4) with type II and III functional responses and small immigration (Figs 3–4). The resulting dynamics of the LV systems (4) with type II functional responses and a fixed number of immigrants on the predator or prey population stabilizes the system (Fig. 3a–d, *c,d* = 0.01), compared to the result of the LV systems (4) without immigrants which destabilizes the system (Fig. 3e, *c,d* = 0, refer to Supplementary Text in the Supplementary Information for the stability analysis). Moreover, the resulting dynamics of the LV systems (4) with type III functional responses and with/without small immigrants on the predator or prey population stabilizes the system (Fig. 4).

**Table 1:** Nature of equilibria of Lotka-Volterra system (4) (refer to Supplementary information for the analytical solution).

Model	Without immigrants	Small immigration			
		*C*(*x*) = *c*; *D*(*y*) = 0	*C*(*x*) = *c*/*x*; *D*(*y*) = 0	*C*(*x*) = 0; *D*(*y*) = *d*	*C*(*x*) = 0; *D*(*y*) = *d*/*y*
Type I (Linear) $$\{\begin{array}{c}\frac{{\rm{d}}x}{dt}=rx-axy+C(x)\\ \frac{{\rm{d}}y}{dt}=bxy-my+D(y)\end{array}$$	Unstable, limit cycle exists	Stable coexistence	Stable coexistence	Stable coexistence	Stable coexistence
Type II (Hyperbolic) $$\{\begin{array}{c}\frac{{\rm{d}}x}{dt}=rx-\frac{axy}{1+hx}+C(x)\\ \frac{{\rm{d}}y}{dt}=\frac{bxy}{1+hx}-my+D(y)\end{array}$$	Unstable	Locally asymptotically stable*	Locally asymptotically stable*	Locally asymptotically stable*	Locally asymptotically stable*
Type III (Sigmoid functional response) $$\{\begin{array}{c}\frac{{\rm{d}}x}{dt}=rx-\frac{a{x}^{2}y}{1+h{x}^{2}}+C(x)\\ \frac{{\rm{d}}y}{dt}=\frac{b{x}^{2}y}{1+h{x}^{2}}-my+D(y)\end{array}$$	Locally asymptotically stable (*β* > 0)	Locally asymptotically stable*	Locally asymptotically stable*	Locally asymptotically stable*	Locally asymptotically stable*

$\beta =(rb-hmr)$. *Note that the characteristic equation is given by ${{\rm{\lambda }}}^{2}-{\rm{Tr}}(J){\rm{\lambda }}+{\rm{Det}}(J)=0$. If ${\rm{Tr}}(J) < 0$ and ${\rm{Det}}(J) > 0$ where *J* is the Jacobian matrix, the steady state is locally asymptotically stable.

## Discussions

Population persistence and extinction are the extremes of population dynamics^5^. The classical LV systems show that periodic orbital relationship between the populations of prey and predator cannot be eliminated over time. Note that population of the predators will collapse if the prey becomes extinct. However, this does not imply that the prey will also die out if the population of the predators collapses. Instead, prey population will continuously persist over time and will most likely proliferate. The only other solution of the LV systems is cyclic oscillation and we cannot find other stable convergence in the classical LV systems. Nonetheless, the stable coexistence of both the prey and predator populations can often be found in predator-prey systems in the wild^10,15–17^. The current findings show that very few migrants either in prey or predator yield asymptotic stability in the LV systems. Some natural and persistent prey-predator systems may be attributed to a very small number of immigrants in the LV systems regardless of prey or predator.

The biological meaning of immigrants is different when we add *c* (or *d*), and when *c*/*x* (or *d*/*y*) is added. In the former case, a small number of immigrants is added constantly to the population in every generation, which can happen if the habitat is attractive to the immigrants based on habitat quality. In the latter case, the number of immigrants changes depending on the current population. Fewer individuals immigrate if the current population is already high, which is a realistic scenario since carrying capacity can limit immigration. Their biological interpretations are thus different, but both cases are qualitatively the same with both resulting to asymptotic stability (Eqs 1–4).

Very small immigration into either prey or predator population acts as a stabilizing factor to the LV systems (Figs 1–4). Adding positive immigration will average out all fluctuations in both the population of prey and predators. Note also from the results that a positive immigration factor is enough to change the quality of the population dynamics of the predator-prey model. This paper may imply that cyclic populations can be stabilized by adding few immigrations into them. In most natural populations, there are at least a few immigrants over time. These small numbers of immigrating species are sufficient for asymptotic stability in the prey-predator systems. This may explain some observed stability in prey-predator systems found in nature.

## Electronic supplementary material

Supplementary Information
