# Supplementary material for: Asymptotic stability of a modified Lotka-Volterra model with small immigrations
Source: Sci Rep. 2018 May 4;8:7029. doi: 10.1038/s41598-018-25436-2 (PMC5935694; doi:10.1038/s41598-018-25436-2)
Supplement: Supplementary file 1 — Supplementary Information [file 41598_2018_25436_MOESM1_ESM.pdf]

Supplementary Information for
Asymptotic stability of a modified Lotka-Volterra model with small immigrations

Takeru Tahara, Maica Krizna Areja Gavina, Takenori Kawano, Jerrold M. Tubay, Jomar F. Rabajante, Hiromu Ito, Satoru Morita, Genki Ichinose, Takuya Okabe, Tatsuya Togashi, Kei-ichi Tainaka, Akira Shimizu, Takashi Nagatani, Jin Yoshimura

Supplementary Tables.

Supplementary Table S1. Summary of the state variables and model parameters.

Variable/Parameter	Description
x	prey population
y	predator population
r	reproduction rate of prey
b	birth rate of predator for each prey captured
a	rate at which predators destroy prey
m	mortality rate of predators
c	small positive immigration factor into prey population
d	small positive immigration factor into predator population
γ	rate of amplitude decay
h	functional response coefficient which involve handling time etc.
J	Jacobian matrix
**Tr(*J*)**	Trace of J
**Det(*J*)**	Determinant of J

Supplementary Table S2. The conditions for the stability of the Lotka-Volterra model with three types of functional responses and prey immigrants. The characteristic equation is given by $\lambda^2 - \mathrm{Tr}(J)\lambda + \mathrm{Det}(J) = 0$. If $\mathrm{Tr}(J) < 0$ and $\mathrm{Det}(J) > 0$, the steady state is locally asymptotically stable.

Model	Small immigration
	***C*(*x*)=*c*, *D*(*y*)=0**
Type I (Linear) $\begin{cases} \frac{dx}{dt} = rx - axy + C(x) \\ \frac{dy}{dt} = bxy - my + D(y) \end{cases}$	$x^* = \frac{m}{b}, y^* = \frac{bc + mr}{am}$ $\mathrm{Tr}(J) = -\frac{bc}{m}$ $\mathrm{Det}(J) = bc + mr$
Type II (Hyperbolic) $\begin{cases} \frac{dx}{dt} = rx - \frac{axy}{1+hx} + C(x) \\ \frac{dy}{dt} = \frac{bxy}{1+hx} - my + D(y) \end{cases}$	$x^* = \frac{m}{b - hm}, y^* = \frac{b(bc - chm + mr)}{am(b - hm)}$ $\mathrm{Tr}(J) = \frac{-c(b - hm)^2 + hm^2 r}{bm}$ $\mathrm{Det}(J) = \frac{(b - hm)(bc + m(-ch + r))}{b}$
Type III (Sigmoid functional response)	$x^* = \frac{\sqrt{m}}{\sqrt{b - hm}}, y^* = \frac{b(c + \frac{\sqrt{mr}}{\sqrt{b - hm}})}{am}$

$\begin{cases} \frac{dx}{dt} = rx - \frac{ax^2y}{1+hx^2} + C(x) \\ \frac{dy}{dt} = \frac{bx^2y}{1+hx^2} - my + D(y) \end{cases}$	$\mathrm{Tr}(J) = -\frac{\frac{2bc\sqrt{b-hm}}{\sqrt{m}} - 2ch\sqrt{m}\sqrt{b-hm} + br - 2hmr}{b}$<br>$\mathrm{Det}(J) = \frac{2\sqrt{b-hm}(bc\sqrt{m} - chm^{3/2} + m\sqrt{b-hm}r)}{b}$

Supplementary Table S3. The condition for the stability of the Lotka-Volterra model with three types of functional responses and constant prey immigrants. The characteristic equation is given by $\lambda^2 - \mathrm{Tr}(J)\lambda + \mathrm{Det}(J) = 0$. If $\mathrm{Tr}(J) < 0$ and $\mathrm{Det}(J) > 0$, the steady state is locally asymptotically stable.

**Model**	**Small immigration**
	***C*(*x*)=*c*/*x*, *D*(*y*)=0**
Type I (Linear)<br>$\begin{cases} \frac{dx}{dt} = rx - axy + C(x) \\ \frac{dy}{dt} = bxy - my + D(y) \end{cases}$	$x^* = \frac{m}{b}, y^* = \frac{b^2c + m^2r}{am^2}$<br>$\mathrm{Tr}(J) = -\frac{2b^2c}{m^2}$<br>$\mathrm{Det}(J) = \frac{b^2c}{m} + mr$
Type II (Hyperbolic)<br>$\begin{cases} \frac{dx}{dt} = rx - \frac{axy}{1+hx} + C(x) \\ \frac{dy}{dt} = \frac{bxy}{1+hx} - my + D(y) \end{cases}$	$x^* = \frac{m}{b-hm}, y^* = \frac{b(b^2c - 2bchm + ch^2m^2 + m^2r)}{am^2(b-hm)}$<br>$\mathrm{Tr}(J) = \frac{-ad(b + h(r-m)) + hmr^2}{br - adh}$<br>$\mathrm{Det}(J) = \frac{(b-hm)r(mr-ad)}{br-adh}$
Type III (Sigmoid functional response)<br>$\begin{cases} \frac{dx}{dt} = rx - \frac{ax^2y}{1+hx^2} + C(x) \\ \frac{dy}{dt} = \frac{bx^2y}{1+hx^2} - my + D(y) \end{cases}$	$x^* = \frac{\sqrt{m}}{\sqrt{b-hm}}, y^* = \frac{b(bc + m(-ch + r))}{am^{3/2}\sqrt{b-hm}}$<br>$\mathrm{Tr}(J) = c(5h - \frac{3b}{m} - \frac{2h^2m}{b}) + (-1 + \frac{2hm}{b})r$<br>$\mathrm{Det}(J) = \frac{2(b-hm)(bc + m(-ch+r))}{b}$

Supplementary Table S4. The condition for the stability of the Lotka-Volterra model with three types of functional responses and predator immigrants. The characteristic equation is given by $\lambda^2 - \mathrm{Tr}(J)\lambda + \mathrm{Det}(J) = 0$. If $\mathrm{Tr}(J) < 0$ and $\mathrm{Det}(J) > 0$, the steady state is locally asymptotically stable.

**Model**	**Small immigration**
	C*(*x*)=0, *D*(*y*)=*d
Type I (Linear)<br>$\begin{cases} \frac{dx}{dt} = rx - axy + C(x) \\ \frac{dy}{dt} = bxy - my + D(y) \end{cases}$	$x^* = \frac{mr - ad}{br}, y^* = \frac{r}{a}$<br>$\mathrm{Tr}(J) = -\frac{ad}{r}$<br>$\mathrm{Det}(J) = mr - ad$
Type II (Hyperbolic)	$x^* = \frac{mr - ad}{(b-hm)r}, y^* = \frac{br - adh}{a(b-hm)}$

$\begin{cases} \frac{dx}{dt} = rx - \frac{axy}{1+hx} + C(x) \\ \frac{dy}{dt} = \frac{bxy}{1+hx} - my + D(y) \end{cases}$	$\mathrm{Tr}(J) = \frac{-c(b-hm)^2(2b-hm) + hm^3 r}{bm^2}$<br>$\mathrm{Det}(J) = \frac{(b-hm)(b^2c - 2bchm + m^2(ch^2+r))}{bm}$
Type III (Sigmoid functional response)<br>$\begin{cases} \frac{dx}{dt} = rx - \frac{ax^2y}{1+hx^2} + C(x) \\ \frac{dy}{dt} = \frac{bx^2y}{1+hx^2} - my + D(y) \end{cases}$	$x^* = \frac{-ad + B}{2(b-hm)r}, y^* = \frac{ad(b-2hm) + bB}{2am(b-hm)}$<br>$\mathrm{Tr}(J) = \frac{a^2d^2(b-2hm) - 2b(b-2hm)r^3 - ad(b+2hr)B}{2(a^2d^2h + b^2r^2)}$<br>$\mathrm{Det}(J) = \frac{r(a^2bd^2 + 4bm(b-hm)r^2 - ad(b-2hm)B)}{2(a^2d^2h + b^2r^2)}$

$A = \sqrt{4a^2dh(-b+hm) + b^2r^2}, B = \sqrt{a^2d^2 + 4m(b-hm)r^2}$

Supplementary Table S5. The condition for the stability of the Lotka-Volterra model with three types of functional responses and constant predator immigrants. The characteristic equation is given by $\lambda^2 - \mathrm{Tr}(J)\lambda + \mathrm{Det}(J) = 0$. If $\mathrm{Tr}(J) < 0$ and $\mathrm{Det}(J) > 0$, the steady state is locally asymptotically stable.

**Model**	**Small immigration**
	C*(*x*)=0, *D*(*y*)=*d/y
Type I (Linear)<br>$\begin{cases} \frac{dx}{dt} = rx - axy + C(x) \\ \frac{dy}{dt} = bxy - my + D(y) \end{cases}$	$x^* = \frac{mr^2 - a^2d}{br^2}, y^* = \frac{r}{a}$<br>$\mathrm{Tr}(J) = -\frac{2a^2d}{r^2}$<br>$\mathrm{Det}(J) = mr - \frac{a^2d}{r}$
Type II (Hyperbolic)<br>$\begin{cases} \frac{dx}{dt} = rx - \frac{axy}{1+hx} + C(x) \\ \frac{dy}{dt} = \frac{bxy}{1+hx} - my + D(y) \end{cases}$	$x^* = \frac{\sqrt{m}}{\sqrt{b-hm}}, y^* = \frac{b(bc + m(-ch+r))}{am^{3/2}\sqrt{b-hm}}$<br>$\mathrm{Tr}(J) = -\frac{2\left(2a^2d(b-hm)(2b+h(-2m+r)) + hmr^2(-br+A)\right)}{(-br+A)^2}$<br>$\mathrm{Det}(J) = -\frac{4(b-hm)r\left(bmr^2(br-A) + 2a^2d(b-hm)(-2hmr+A)\right)}{(-br+A)^3}$
Type III (Sigmoid functional response)<br>$\begin{cases} \frac{dx}{dt} = rx - \frac{ax^2y}{1+hx^2} + C(x) \\ \frac{dy}{dt} = \frac{bx^2y}{1+hx^2} - my + D(y) \end{cases}$	$x^* = \frac{-(b-2hm)r + A}{2h(b-hm)r}, y^* = \frac{br + A}{2a(b-hm)}$<br>$\mathrm{Tr}(J) = \frac{-4a^2d(b-hm)(2b+h(-2m+r)) + 2hmr^2(br+A)}{(br+A)^2}$<br>$\mathrm{Det}(J) = \frac{4(b-hm)r(bmr^2(br+A) - 2a^2d(b-hm)(2hmr+A))}{(br+A)^3}$

$A = \sqrt{4a^2dh(-b+hm) + b^2r^2}, B = \sqrt{a^2d^2 + 4m(b-hm)r^2}$

Supplementary Text. Determining the asymptotic stability of the LV system with type I functional response and few immigrants (or with few migrants). Also, the asymptotic stability analysis for LV system with type II and III functional responses but without immigrants.

I. Asymptotic stability of the LV system with type I functional response and prey immigrants (or migrants)

Here we investigate the stability of a steady state solution

$$(x^*, y^*) = \left(\frac{m}{b}, \frac{mr+bc}{am}\right)$$

of modified Lotka-Volterra equations with a positive prey immigration factor ***c*** given by

$$\begin{cases} \dfrac{dx}{dt} = rx - axy + c \\ \dfrac{dy}{dt} = bxy - my \end{cases} . \qquad (1)$$

The system has two steady-state solutions: $\left(\frac{-c}{r}, 0\right)$ and $(x^*, y^*) = \left(\frac{m}{b}, \frac{mr+bc}{am}\right)$. The Jacobian matrix of LV system (1) is given by

$$J = \begin{bmatrix} r - ay & -ax \\ by & bx - m \end{bmatrix}.$$

The characteristic equation of the Jacobian matrix *J* at point $(x^*, y^*) = \left(\frac{m}{b}, \frac{mr+bc}{am}\right)$ is

$$\lambda^2 + \frac{bc}{m}\lambda + (bc + mr) = 0 .$$

The general solution of this equation is rotational motion in a (x, y) plane. The equation has eigenvalues $\pm i\omega - \gamma$, where

$$\omega = \sqrt{mr + bc - \frac{b^2c^2}{4m^2}} ,$$

and

$$\gamma = \frac{bc}{2m} .$$

If the former is a real quantity, it represents the angular frequency of rotation. The latter γ is the rate of amplitude decay. The steady state (x^*, y^*) is stable if $\gamma > 0$ and unstable if $\gamma < 0$.

Similarly, when a positive migration rate c is added to the prey equation,

$$\begin{cases} \dfrac{dx}{dt} = rx - axy - c \\ \dfrac{dy}{dt} = bxy - my \end{cases} . \qquad (2)$$

For sufficiently small values of c, we obtain the decay rate

$$\gamma = \frac{-bc}{2m}.$$

Hence, migrations of the prey species destabilize the system.

II. Asymptotic stability of the LV system with type I functional response and predator immigrants (or migrants)

Here we investigate the stability of a steady state solution

$$(x^*, y^*) = \left(\frac{mr - ad}{br}, \frac{r}{a} \right)$$

of modified Lotka-Volterra equations with a positive predator immigration factor ***d*** given by

$$\begin{cases} \dfrac{dx}{dt} = rx - axy \\ \dfrac{dy}{dt} = bxy - my + d \end{cases} . \qquad (3)$$

The system has two steady-state solutions: $\left(0, \dfrac{d}{m}\right)$ and $(x^*, y^*) = \left(\dfrac{mr - ad}{br}, \dfrac{r}{a} \right)$. The Jacobian matrix of LV system (3) is given by

$$J = \begin{bmatrix} r - ay & -ax \\ by & bx - m \end{bmatrix}.$$

The characteristic equation of the Jacobian matrix J at point $(x^*, y^*) = \left(\dfrac{mr - ad}{br}, \dfrac{r}{a} \right)$ is

$$\lambda^2 + \frac{ad}{r}\lambda + (mr - ad) = 0 .$$

The general solution of this equation is rotational motion in a (x, y) plane. The equation has eigenvalues $\pm i\omega - \gamma$, where

$$\omega = \sqrt{ad - mr - \frac{a^2 d^2}{4r^2}}$$

and

$$\gamma = \frac{ad}{2r}.$$

If the former is a real quantity, it represents the angular frequency of rotation. The latter γ is the rate of amplitude decay. The steady state (x^*, y^*) is stable if $\gamma > 0$ and unstable if $\gamma < 0$.

Similarly, when a positive migration rate d is added to the predator equation,

$$\begin{cases} \frac{dx}{dt} = rx - axy \\ \frac{dy}{dt} = bxy - my - d \end{cases}. \tag{4}$$

For sufficiently small values of d, we obtain the decay rate

$$\gamma = \frac{-ad}{2r}.$$

Hence, migrations of the predator species destabilize the system.

In general, when a positive constant rate c is added to the prey equation and positive constant rate d is added to the predator equation,

$$\begin{cases} \frac{dx}{dt} = rx - axy + c \\ \frac{dy}{dt} = bxy - my + d \end{cases}. \tag{5}$$

For sufficiently small values of c and d, we obtain the decay rate

$$\gamma = \frac{bc}{2m} + \frac{ad}{2r}.$$

In conclusion, immigration of the predator $(d > 0)$ has the same stabilization effect as that of the prey $(c > 0)$. Conversely, migration of both species destabilizes the system.

III. Asymptotic stability of the LV system with type I functional response and few prey immigrants (or migrants)

Here we investigate the stability of the steady state solution

$$(x^*, y^*) = \left(\frac{m}{b}, \frac{m^2 r + b^2 c}{m^2 a} \right)$$

of modified Lotka-Volterra equations with a positive constant immigration factor $\boldsymbol{c}$ given by

$$\begin{cases} \frac{dx}{dt} = rx - axy + \frac{c}{x} \\ \frac{dy}{dt} = bxy - my \end{cases}. \tag{6}$$

The system has one steady-state solution: $(x^*, y^*) = \left(\frac{m}{b}, \frac{m^2 r + b^2 c}{m^2 a}\right)$. The Jacobian matrix of system (6) is given by

$$J = \begin{bmatrix} r - ay - \frac{c}{x^2} & -ax \\ by & bx - m \end{bmatrix}.$$

The characteristic equation of the Jacobian matrix J at point $(x^*, y^*) = \left(\frac{m}{b}, \frac{m^2 r + b^2 c}{m^2 a}\right)$ is

$$\lambda^2 + \frac{2b^2 c}{m^2}\lambda + \left(mr + \frac{b^2 c}{m}\right) = 0$$

The general solution of this equation is rotational motion in a (x, y) plane. The equation has eigenvalues $\pm i\omega - \gamma$, where

$$\omega = \sqrt{mr + \frac{b^2 c}{m} - \frac{b^4 c^2}{m^4}},$$

and

$$\gamma = \frac{b^2 c}{m^2}.$$

If the former is a real quantity, it represents the angular frequency of rotation. The latter γ is the rate of amplitude decay. The steady state (x^*, y^*) is stable if $\gamma > 0$ and unstable if $\gamma < 0$.

Similarly, when a positive constant migration rate c is added to the prey equation,

$$\begin{cases} \frac{dx}{dt} = rx - axy - \frac{c}{x}, \\ \frac{dy}{dt} = bxy - my. \end{cases} \tag{7}$$

For sufficiently small values of c, we obtain the decay rate

$$\gamma = \frac{-b^2 c}{m^2}.$$

Hence, constant migrations of the prey species destabilize the system.

IV. Asymptotic stability of the LV system with type I functional response and few predator immigrants (or migrants)

Here we investigate the stability of a steady state solution

$$\left(x^*, y^*\right) = \left(\frac{mr^2 - a^2 d}{br^2}, \frac{r}{a}\right)$$

of modified Lotka-Volterra equations with a positive constant immigration factor ***d*** given by

$$\begin{cases} \dfrac{dx}{dt} = rx - axy, \\ \dfrac{dy}{dt} = bxy - my + \dfrac{d}{y}. \end{cases} \tag{8}$$

The system has three steady-state solutions: $\left(0, \pm\sqrt{\frac{d}{m}}\right)$ and $\left(x^*, y^*\right) = \left(\frac{mr^2 - a^2 d}{br^2}, \frac{r}{a}\right)$. The Jacobian matrix of system (8) is given by

$$J = \begin{bmatrix} r - ay & -ax \\ by & bx - m - \dfrac{d}{y^2} \end{bmatrix}.$$

The characteristic equation of the Jacobian matrix J at point $\left(x^*, y^*\right) = \left(\frac{mr^2 - a^2 d}{br^2}, \frac{r}{a}\right)$ is

$$\lambda^2 + \frac{2a^2 d}{r^2}\lambda + \left(\frac{mr^2 - a^2 d}{r}\right) = 0.$$

The general solution of this equation is rotational motion in a (x, y) plane. The equation has eigenvalues $\pm i\omega - \gamma$, where

$$\omega = \sqrt{\frac{-a^4 d^2}{r^4} + mr - \frac{a^2 d}{r}},$$

and

$$\gamma = \frac{a^2 d}{r^2}.$$

If the former is a real quantity, it represents the angular frequency of rotation. The latter γ is the rate of amplitude decay. The steady state (x^*, y^*) is stable if $\gamma > 0$ and unstable if $\gamma < 0$.

Similarly, when a positive migration rate d is added to the predator equation,

$$\begin{cases} \dfrac{dx}{dt} = rx - axy, \\ \dfrac{dy}{dt} = bxy - my - \dfrac{d}{y}. \end{cases} \tag{9}$$

For sufficiently small values of d, we obtain the decay rate

$$\gamma = \frac{-a^2 d}{r^2}.$$

Hence, constant migrations of the predator species destabilize the system.

V. Asymptotic stability of the LV system with type II functional response (without immigrants)

Here we investigate the stability of a steady state solution

$$(x^*, y^*) = \left(\frac{m}{b-hm}, \frac{br}{ab-ahm}\right)$$

of modified Lotka-Volterra equations with a positive prey immigration factor ***c*** given by

$$\begin{cases} \dfrac{dx}{dt} = rx - \dfrac{axy}{1+hx}, \\ \dfrac{dy}{dt} = \dfrac{bxy}{1+hx} - my. \end{cases} \tag{10}$$

The system has two steady-state solutions: $(0,0)$ and $(x^*, y^*) = \left(\frac{m}{b-hm}, \frac{br}{ab-ahm}\right)$. The Jacobian matrix of LV system (10) is given by

$$J = \begin{bmatrix} r - \dfrac{ay}{(1+hx)^2} & \dfrac{-ax}{1+hx} \\ \dfrac{by}{(1+hx)^2} & \dfrac{bx}{1+hx} - m \end{bmatrix}.$$

The characteristic equation of the Jacobian matrix J at point $(x^*, y^*) = \left(\frac{m}{b-hm}, \frac{br}{ab-ahm}\right)$

is $\lambda^2 - \frac{hmr}{b}\lambda + \left(mr - \frac{hm^2 r}{b}\right) = 0$

The general solution of this equation is rotational motion in a (x, y) plane. The equation has eigenvalues $\pm i\omega - \gamma$, where

$$\omega = \left(\frac{b^4 - 4b^3hm + 6b^2h^2m^2 - 4bh^3m^3 + h^4m^4}{2b^4}\right)\sqrt{\frac{b^6 mr\left(4b^2 - 4bhm - h^2mr\right)}{(b-hm)^8}},$$

and

$$\gamma = \frac{-hmr}{2b}$$

If the former is a real quantity, it represents the angular frequency of rotation. The latter γ is the rate of amplitude decay. The steady state (x^*, y^*) is stable if $\gamma > 0$ and unstable if $\gamma < 0$.

VI. Asymptotic stability of the LV system without immigrants

Here we investigate the stability of the steady state solutions

$$\left(x^*, y^*\right) = \left(\pm\sqrt{\frac{m}{b-hm}}, \pm\frac{br}{a\sqrt{m\left(b-hm\right)}} \right)$$

of modified Lotka-Volterra equations with a positive prey immigration factor ***c*** given by

$$\begin{cases} \dfrac{dx}{dt} = rx - \dfrac{ax^2 y}{1+hx^2}, \\ \dfrac{dy}{dt} = \dfrac{bx^2 y}{1+hx^2} - my. \end{cases} \tag{11}$$

The system has three steady-state solutions: $\left(0,0\right)$ and

$\left(x^*, y^*\right) = \left(\pm\sqrt{\frac{m}{b-hm}}, \pm\frac{br}{a\sqrt{m\left(b-hm\right)}} \right)$. The Jacobian matrix of LV system (11) is given by

$$J = \begin{bmatrix} r - \dfrac{2axy}{\left(1+hx^2\right)^2} & \dfrac{-ax^2}{1+hx^2} \\ \dfrac{2bxy}{\left(1+hx^2\right)^2} & \dfrac{bx^2}{1+hx^2} - m \end{bmatrix}.$$

The characteristic equation of the Jacobian matrix J at point

$\left(x^*, y^*\right) = \left(\pm\sqrt{\frac{m}{b-hm}}, \pm\frac{br}{a\sqrt{m\left(b-hm\right)}} \right)$ is $\lambda^2 + \left(r - \frac{2hmr}{b}\right)\lambda + \left(2mr - \frac{2hm^2 r}{b}\right) = 0$

The general solution of this equation is rotational motion in a (x, y) plane. The equation has eigenvalues $\pm i\omega - \gamma$, where

$$\omega = \left(\frac{b^4 - 4b^3hm + 6b^2h^2m^2 - 4bh^3m^3 + h^4m^4}{2b^4}\right)\sqrt{\frac{b^6 r\left(\begin{matrix} 8b^2m - 8bhm^2 - b^2 r \\ +4bhmr - 4h^2m^2r \end{matrix}\right)}{\left(b-hm\right)^8}},$$

and

$$\gamma = \frac{rb - hmr}{2b}.$$

If the former is a real quantity, it represents the angular frequency of rotation. The latter γ is the rate of amplitude decay. The steady state (x^*, y^*) is stable if $\gamma > 0$ and unstable if $\gamma < 0$.
